# Interrogating imaginary optical force by the complex Maxwell stress tensor theorem

**DOI:** 10.1038/s41377-022-01049-3

**Published:** 2023-01-11

**Authors:** Jinwei Zeng, Jian Wang

**Affiliations:** 1 Wuhan National Laboratory for Optoelectronics and School of Optical and Electronic Information, Huazhong University of Science and Technology, Wuhan, 430074 Hubei China; 2 Optics Valley Laboratory, Wuhan, 430074 Hubei China

**Keywords:** Micro-optics, Nanoparticles

## Abstract

The complex Maxwell stress tensor theorem has been developed to relate the imaginary optical force, reactive strength of canonical momentum and total optical force of a nanoparticle, which is essential to perfect optical force efficiency.

Material properties can be interrogated through monitoring light-matter interactions. Conventionally, the photo-detector (PD) has been served as a fundamental device to detect photon energy, which can reveal the energy-transfer information associated to a certain light-matter interaction scenery. To relate the transferred energy and the local electromagnetic field near a particle, the Poynting theorem has been developed based on the energy conservation law as an important foundation in the electromagnetic theory. Note that the Poynting vector may simultaneously include the real part to describe the active power, and the imaginary part to describe the reactive (stored) power. Therefore, together, they make the complex Poynting theorem (CPT) to establish a faithful connection between the optical energy and the optical field^1^.

Energy is not the only quantity that must be conserved during light-matter interaction sceneries. Similar to energy, the momenta, as well as the means to change them, i.e., the forces, are also conservative. Accordingly, to relate the change of momentum and the exerted electromagnetic force on a particle, the Maxwell stress tensor (MST) theorem has been developed as an important theoretical foundation to understand the nature of optical forces^1^. While conventional optics often rely on photon energy detection and exploitation, the optical force provides new and special perspectives to interpret light-matter interactions: not only because the force is a vector that can carry more information than the energy as a scalar, but also due to the special manipulability of the force to control and move the particles. Especially, thanks to the recent fast-growing nanotechnology, optical force has become a promising field that inspires many important applications, such as the photo-induced force microscopy^2–4^, optical tweezers^5–7^, chiral separation^8^ etc. Notably, the 2018 Nobel prize of physics has been awarded to Authur Ashkin for his distinguished contribution in optical tweezers, as the exemplary fruition of optical forces^9,10^.

As already widely applied in practice, currently established MST theorem mainly considers the real part of the optical force, i.e., the real (time-averaged) Lorentz force (RLF), the momentum flux created by the RLF, and the density of the flow as the real part of the MST (RMST). These parameters are all real and can be accurately characterized by time-averaged experiment. However, compared to the complex Poynting theorem, current MST seems to have missed a critical piece of puzzle, i.e., the imaginary part of the force and the momentum, so that it only tells half a story about the force-momentum relation.

To tell the complete story, in a newly published paper in Light: Science & Applications, Manuel Nieto-Vesperinas from the Instituto de Ciencia de Materiales de Madrid, CSIC, Spain, and Xiaohao Xu from the State Key Laboratory of Transient Optics and Photonics and Xi’an Institute of Optics and Precision Mechanics, Chinese Academy of Sciences, China, have proposed the complex MST (CMST) theorem to refill the missing imaginary part^11^. As they have discovered, much similar to the CPT, the imaginary part of the CMST is related to the reactive orbital or canonical momentum (ROM) which is stored in the nearfield of the illuminated particle, while the RMST dictates the radiated momentum flow to the farfield, as illustrated in Fig. 1. While the IMST builds up the ROM in the nearfield of the particle, the imaginary part of the Lorentz force (ILF) can be therefore defined and determined. Furthermore, the conservative nature of CMST demands the ILF and the stored ROM to counteract with the RLF: i.e., a larger ILF and ROM storage makes a smaller radiated RLF to the farfield.

**Fig. 1:**
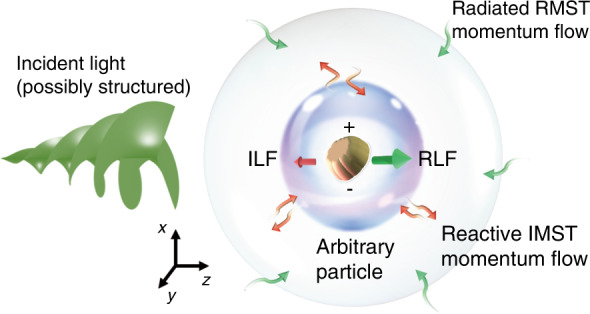
Schematic view of the CMST concept. The size of the particle, nearfield and farfield region are adjusted for the ease of reading. Incident light, possibly having special field structures, illuminates and polarizes a particle. Such interaction induces both real and imaginary optical force, i.e., ILF and RLF that results in the reactive IMST momentum flow and radiated RMST momentum flow, respectively. The pair of ILF and RLF, and the pair of IMST and RMST, exhibit counter-act effect which means the smaller the ILF and IMST, the bigger the RLF and RMST

The above observation can lead to an important principle to optimize optical force efficiency in practice. Since the RLF is actually the detectable time-averaged optical force in typical optical force applications, it means that to achieve an ideal optical force efficiency in optical tweezers, optical antennas, or photo-induced force microscopy etc, one needs to accordingly suppress the ROM and the ILF in the corresponding system. For given light-matter interaction sceneries, as illustrated in this work, the ROM and the CMST of a nano-particle can be calculated through the effective polarizabilities in the multipole modal. To verify this principle, the authors have illustrated typical light-matter interaction sceneries as examples, including linearly or circularly polarized plane wave illuminating on plasmonic or dielectric particles. Especially, in the case of a high-index and low-loss Si nano-structure with magnetic Mie resonance, the authors make theoretical derivation showing suppressed ILF can induce significant radiated RLF with enhanced quality factor near the resonant region, which agrees well with the experimental characterization acquired by the photo-induced force microscopy reported previously^12^.

Looking forward, while the CMST theorem establishes an important foundation of optical force theory, it also fosters inspirations to future possibilities in optical force applications. As illustrated in Fig. 2, seeing the big picture of the light-matter interaction sceneries, both the illumination light and the particle under investigation can be more complex than the conventional fundamental Gaussian beam and the simple isotropic materials as discussed in this work. On the illumination side, the light source can have an inhomogeneous field structure such as the Hermite-Gaussian (HG) beam, orbital angular momentum (OAM) beam, and vector beam^13–15^ etc., also known as the structured light. On the particle side, it can have anisotropy, chirality, optically magnetic responsibility, or even be non-reciprocal under external excitation, to exhibit various forms of asymmetry^16^. Such property can either be introduced based on the material itself, or can be artificially bestowed through special nano-structures (e.g., metamaterials, metasurfaces), also known as the structured matter. The combination of structured light and structured matter can induce special multipole modals to selectively exhibit certain optical properties^17,18^. And the optical force as an unique detection or manipulation method, can further decorate the picture.

**Fig. 2:**
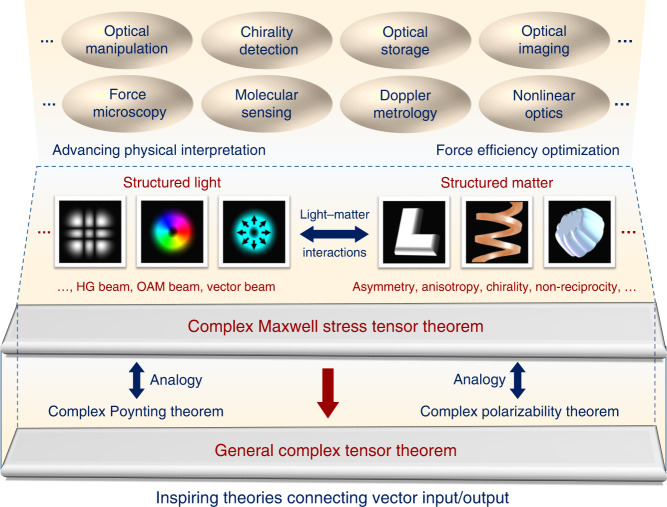
Future vision of the CMST theory for fundamental physics and advanced applications

It is foreseenable that the proposed CMST theory can give rise to exciting inspirations for both fundamental physics and advanced optical force applications. On the physics side, the CMST theorem presents the relation between real and imaginary part of the MST in analogy of the CPT and the complex polarizability theorem. This principle may be naturally and reasonably extended to more general tensor theorem, i.e., any physical tensors connecting two conservative physical quantities that yet have the complex value definition. On the application side, optical force is already or potentially applied to optical manipulation, chirality detection/separation, optical storage and reading, optical imaging, force microscopy, molecular chemical sensing, optical Doppler metrology, nonlinear optics, etc.^8,12,19–23^. The CMST theorem will play an essential role to analyze and optimize these applications, towards advanced physical interpretations with ideal force detecting presentations.
